# Kinase function of TgTKL1 is essential for its role in *Toxoplasma* propagation and pathogenesis

**DOI:** 10.1128/msphere.00779-24

**Published:** 2024-10-30

**Authors:** Dima Hajj Ali, Ramu Anandakrishnan, Vern B. Carruthers, Rajshekhar Y. Gaji

**Affiliations:** 1 Department of Biomedical Sciences and Pathobiology, Virginia-Maryland College of Veterinary Medicine, Blacksburg, Virginia, USA; 2 Department of Biomedical Sciences, Edward Via College of Osteopathic Medicine (VCOM), Blacksburg, Virginia, USA; 3 Department of Microbiology and Immunology, University of Michigan, Ann Arbor, Michigan, USA; Australian National University, Canberra, Australia

**Keywords:** apicomplexan parasites, *Toxoplasma gondii*, kinases

## Abstract

The Tyrosine Kinase-Like (TKL) family of proteins are a set of poorly studied kinases that have garnered attention in recent years for their role in *Toxoplasma* biology. The *Toxoplasma* genome contains eight TKL kinases, of which six have been predicted to be important for parasite propagation. We have previously shown that TgTKL1 is a nuclear kinase that is critical for the parasite lytic cycle and is essential for acute virulence in the animal model. However, the contribution of the kinase domain to the functioning of TgTKL1 was not known. Hence to determine the significance of its catalytic function, we first validated that TgTKL1 is a true kinase using purified recombinant protein. Furthermore, we successfully generated a TgTKL1 kinase mutant strain of *Toxoplasma* via CRISPR-Cas9 gene editing. Our studies revealed that the kinase mutant of TgTKL1 displays defects in parasite growth and host-cell invasion. Additionally, loss of kinase function alters the transcriptomic profile of the parasite, including downregulation of the invasion-related gene, TgSUB1. Importantly, this dysregulation of TgSUB1 expression leads to defects in post-exocytosis processing of micronemal proteins, an event critical for normal host-cell invasion. Furthermore, the TgTKL1 kinase mutant is completely avirulent in the mouse model of acute toxoplasmosis. Since the loss of kinase function leads to phenotypic manifestations seen previously with TgTKL1 knockout parasites, we conclude that kinase activity is important for TgTKL1 function in *Toxoplasma* propagation and virulence.

**IMPORTANCE:**

*Toxoplasma gondii* is a protozoan parasite that can cause life-threatening disease in humans. Hence, identifying key factors required for parasite growth and pathogenesis is important to develop novel therapeutics. We have previously shown that a member of the TKL protein kinase family, TgTKL1, is a plant-like kinase that is required for effective Toxoplasma growth *in vitro* and essential for virulence *in vivo*. Herein, we show that the TgTKL1 is, indeed, a *bona fide* kinase, and loss of its kinase function in the Toxoplasma leads to similar defects seen in parasites with complete loss of TgTKL1. More specifically, the TgTKL1 kinase mutant exhibits defects in parasite growth, host-cell invasion, gene expression profile, and virulence in the animal model. Together, these findings suggest that TgTKL1 is a true kinase, and loss of its kinase activity leads to disruption of TgTKL1 function in *Toxoplasma*.

## INTRODUCTION

*Toxoplasma gondii*, the causative agent of toxoplasmosis, is an obligate intracellular protozoan parasite belonging to the phylum Apicomplexa, comprising important human pathogens including *Plasmodium* spp. causing malaria and *Cryptosporidium* that causes diarrhea in neonates (1–3). It is estimated that about one-third of the human population is infected with *Toxoplasma* (4). However, in the United States, the seropositivity rate ranges from 10% to 12% (5). Human infection of *Toxoplasma* primarily occurs through the oral ingestion of bradyzoite-containing tissue cysts present in contaminated raw or undercooked meat or through the consumption of sporulated oocysts found in food or water contaminated with felines’ feces (4). Additionally, vertical transmission can occur from a pregnant primo-infected mother to the fetus, resulting in congenital toxoplasmosis (6).

Upon infection, the acute form of the parasite (tachyzoite) rapidly disseminates to various organs in the host causing acute infection (4). At this stage, toxoplasmosis is often asymptomatic in over 80% of immunocompetent patients (4). Nevertheless, severe manifestations such as ocular disease and cervical lymphadenopathy may occur (7). Subsequently, with the induction of host immune response, tachyzoites evade the immune system by differentiating into bradyzoites, mainly in the brain and skeletal muscles. These bradyzoites surrounded by a cyst wall are protected from the host immune system and persist for the lifetime of the host (4, 7). The appearance of bradyzoites marks the beginning of chronic toxoplasmosis (8). In immunocompromised individuals or those undergoing immunosuppressive therapy, bradyzoites in the cyst reactivate by differentiating back to tachyzoite stage and can cause fatal encephalitis (7). In pregnant women, primary infection can result in miscarriage, whereas in newborn children, blindness and cognitive impairments may occur (4).

The intracellular lifestyle of *Toxoplasm*a initiates with parasite invasion into host cells, followed by replication within a specialized compartment called the parasitophorous vacuole (PV) through endodyogeny. After successive rounds of division, the parasites egress from host cells, which ultimately results in the destruction of the host-cell (2, 3, 9). Significantly, the pathology associated with *Toxoplasma* infection predominantly emanates from tissue destruction caused by these repetitive cycles of invasion, replication, and egress. Therefore, identifying distinct parasite factors essential for pathogenesis is pivotal for developing novel therapeutic interventions against toxoplasmosis.

In *Toxoplasma,* kinases have been shown to play a critical role in parasite motility, invasion, replication, egress, and survival within the host (3, 10–17). Among these, TKL family kinases have gained attention in recent years, and many of these proteins contain domains that are unique and different from their mammalian counterparts (18, 19). Consequently, defining the role of TKL kinases offers an opportunity not only to understand their role in parasite biology but also allows for development of new therapeutic interventions.

The *Toxoplasma* genome contains eight TKL genes of which six are predicted to be important for parasite propagation *in vitro* (20, 21). TgTKL1 is a plant-like nuclear kinase, and we have previously shown that this protein is important for *Toxoplasma* growth *in vitro*, is involved in gene regulation, and is essential for parasite virulence *in vivo* (18). However, it is not known if the kinase activity of TgTKL1 is, indeed, important for its function. In this report, we provide the evidence that TgTKL1 kinase domain is critical for its function in parasite biology and pathogenesis. More specifically, our findings demonstrate a crucial role for TgTKL1 kinase activity in parasite growth and host-cell invasion. Furthermore, the loss of TgTKL1 catalytic activity results in the downregulation of multiple invasion-related genes, leading to defects in the secretion and processing of micronemal proteins. These results, thus, underscore the significance of TgTKL1 kinase domain in the functioning of this plant-like kinase in *Toxoplasma* biology.

## RESULTS

### TgTKL1 is a true kinase

The protein kinase fold of eukaryotic protein kinases (ePKs) is about 250–300 amino acids in length and consists of two distinct lobes that include conserved motifs (22, 23). The smaller N-lobe is predominantly made of β-sheets, whereas the larger C-lobe is largely made of α-helices (24). Alignment of the TgTKL1 kinase domain sequence with Troponin I-Interacting Kinase (TNNI3K) shows that all the residues typically required for catalytic activity are present in TgTKL1 including the DFG motif (18) (Fig. S1A). Structural modeling of the TgTKL1 kinase domain reveals that the domain shares both N-terminal and C-terminal features typical of ePKs (Fig. S1B).

**Fig 1:**
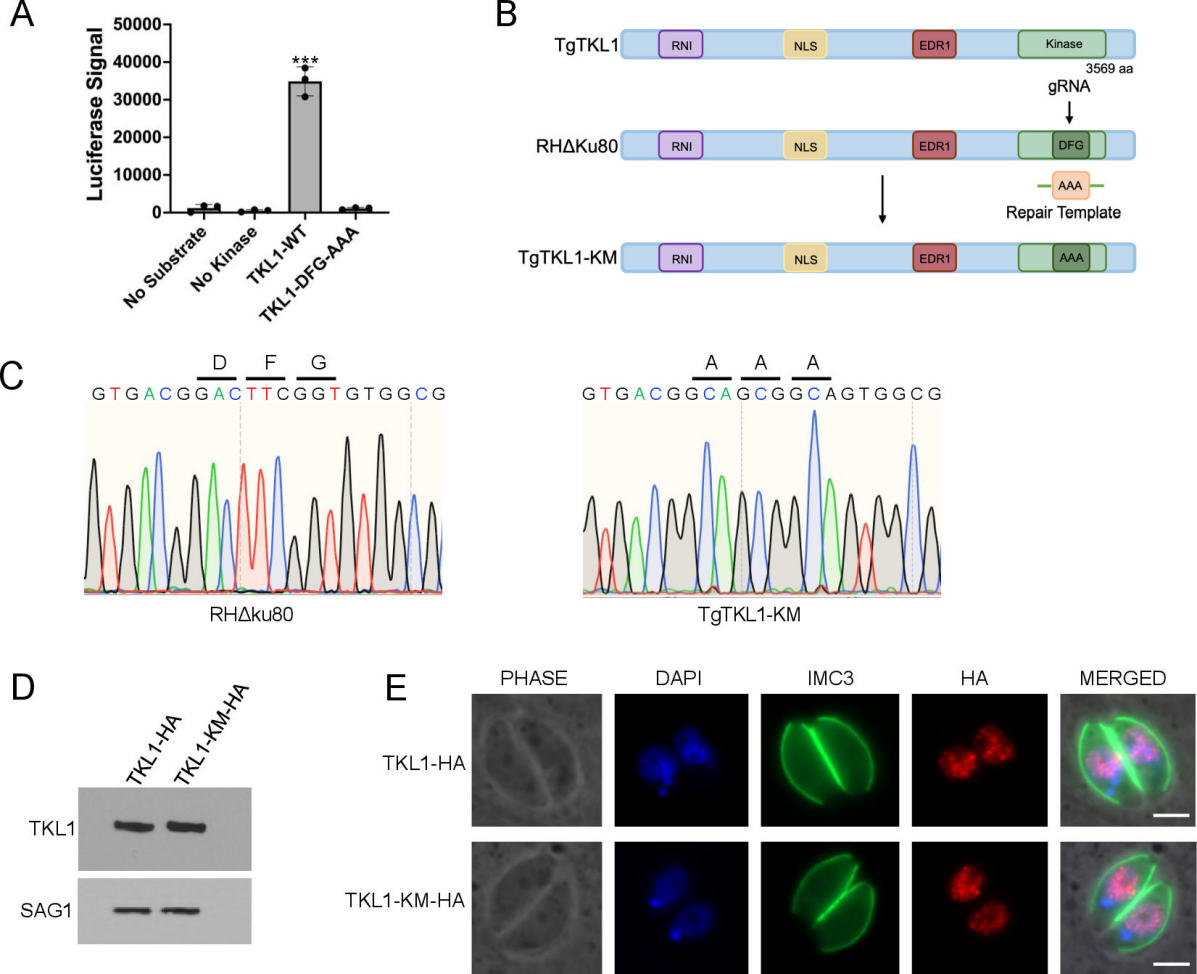
TgTKL1 kinase activity and generation of TgTKL1 kinase mutant. (**A**) Kinase activity of recombinant wild-type TgTKL1 (TKL1-WT) with or without the substrate, myelin basic protein (MBP), and recombinant DFG mutant protein (TKL1-DFG-AAA) with MBP. *n* = 3, Error bars = SD, ***P < 0.001, One-way analysis of variance (ANOVA) using GraphPad Prism. (**B**) Schematic representation illustrating the generation of TgTKL1 kinase mutant strain by replacing the DFG motif with AAA motif using CRISPR/Cas9 technology. (**C**) Sanger sequencing data showing genomic sequence coding for the DFG motif in the wild-type strain (left) and mutant AAA motif in the TgTKL1-KM strain (right). (**D**) Western blot analysis of TgTKL1-HA and TgTKL1-KM-HA parasites with anti-HA antibody. SAG1 is used as the loading control. (**E**) Immunofluorescence analysis of the HA epitope-tagged wild-type TgTKL1-HA and TgTKL1 kinase mutant strain. Wild-type and mutant versions of TgTKL1 were visualized by staining with anti-HA antibody, while IMC3 (green) was used as a marker for the IMC. Scale bar, 2 µm.

To determine if TgTKL1 is a true kinase, we cloned and expressed the kinase domain of TgTKL1 in *E. coli* and purified the recombinant TgTKL1 (Fig. S1C). *In vitro* kinase assay using myelin basic protein (MBP) as a substrate showed that recombinant wild-type TgTKL1 protein exhibits phosphorylation activity, thus indicating that TgTKL1 is an active kinase (Fig. 1A). Since it is well established that DFG motif is essential for kinase function of ePKs (23), we mutated the TgTKL1 DFG motif, purified the resulting TKL1-DFG-AAA recombinant protein, and found that it had no kinase activity (Fig. 1A; Fig. S1C). Together, these findings establish that TgTKL1 is a functional kinase that relies on its DFG motif for catalytic activity.

### Generation of TgTKL1 kinase mutant strain

TgTKL1 protein localizes to the parasite nucleus in *Toxoplasma,* and we have previously shown that this kinase is important for parasite growth *in vitro* and is essential for parasite virulence *in vivo* (18). However, the significance of phosphorylation activity of TgTKL1 in its functioning is not known. The kinase domain of TgTKL1 is located toward the C-terminal region of the protein and is comprised of residues 2659 to 2882 (18). To determine the significance of TgTKL1 catalytic activity to its overall function, we generated a kinase mutant *Toxoplasma* strain by the replacing DFG motif with alanine residues in the RHΔKu80 strain using the CRISPR/Cas9 technology (25) (Fig. 1B). Stable clones were obtained through transformation and selection procedures described in the Materials and Methods followed by limited dilution. Successful mutation of the DFG motif in the TgTKL1 kinase mutant (TgTKL1-KM) was verified by PCR amplification and sequencing (Fig. 1C).

To determine if mutation of the DFG motif affects the expression and localization of TgTKL1, we inserted an HA epitope before the stop codon of TgTKL1 gene of the TgTKL1-KM strain (26). Immunoblotting analysis with anti-HA antibody revealed that TgTKL1 is expressed at similar levels in the kinase mutant and wild-type parasites (Fig. 1D). We also performed immunofluorescence assay (IFA) analysis of wild-type and TgTKL1-KM strain using anti-HA antibody, and the results showed that the mutant TgTKL1 localizes to the parasite nucleus similar to wild-type TgTKL1 (Fig. 1E). These findings suggested that the mutation of the kinase domain did not affect expression or localization of TgTKL1 protein in *Toxoplasma*. Furthermore, we complemented the TgTKL1-KM strain with a wild-type copy of TgTKL1 (Fig. S2) to generate the TgTKL1 kinase mutant complemented strain (TgTKL1-KM-COM) as described in Materials and Methods (18).

### TgTKL1 kinase function is important for parasite attachment to target cells

**Fig 2:**
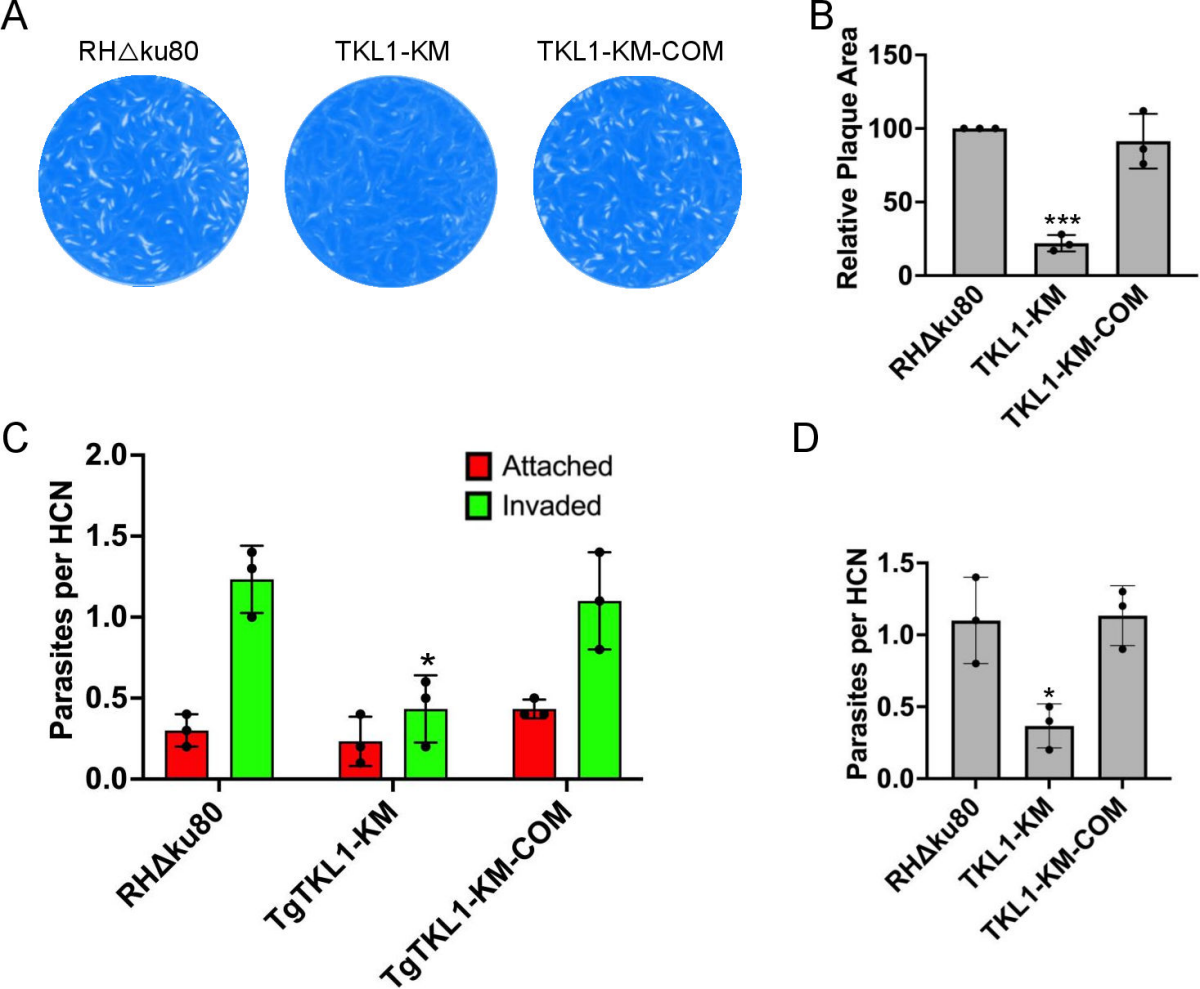
TgTKL1 kinase domain is important for parasite growth *in vitro*. (**A**) Plaque assay examining the growth of wild-type (RHΔKu80), kinase mutant (TgTKL1-KM), and complemented (TgTKL1-KM-COM) strains in HFF cells. Plaques are visible as clear zones on the background of a crystal violet-stained HFF monolayer. (**B**) Quantification of plaque area sizes of wild-type, TgTKL1-KM, and TgTKL1-KM-COM strains. *n* = 3, data represent mean ± standard deviation. ***P < 0.001, One-way ANOVA using GraphPad Prism. (**C**) Red-green invasion assay of *Toxoplasma* tachyzoites after 30 min of incubation with HFF cells. Data represent mean ± standard deviation, *n* = 3 biological replicates. *P < 0.05, One-way ANOVA using GraphPad Prism. HCN, host cell nucleus. (**D**) Host-cell attachment assay. *Toxoplasma* tachyzoites were treated for 10 min with mycalolide B, washed, and then added onto HFF cells. At 30 min post incubation at 37°C, slides were washed, fixed, and stained, and the numbers of attached parasites were quantified. *n* = 3, data show mean ± standard deviation. *P < 0.05, one-way ANOVA using GraphPad Prism. HCN, host cell nucleus.

We next sought to determine the phenotypic consequences of the mutation of the TgTKL1 kinase domain on the lytic cycle of the parasite. To address this question, we performed plaque assays that help to determine parasite propagation ability *in vitro*. We found that compared to wild-type parasites (RHΔku80), the kinase mutant (TgTKL1-KM) showed a significant reduction (78%) in plaque size (Fig. 2A and B), indicating a deficiency in the lytic cycle like that exhibited by the TgTKL1 null mutant parasites (18). Importantly, this reduction in plaque size was restored in the TgTKL1-KM-COM parasite strain. These findings suggest that the kinase activity of TgTKL1 is required for *Toxoplasma* propagation *in vitro*. We also compared plaque-forming ability of TgTKL1 kinase mutant with TgTKL1 null mutants. The results showed that TgTKL1 kinase mutant showed decreased plaque size similar to the null mutant, thus suggesting that loss of kinase function leads to a defect in parasite propagation similar to complete loss of TgTKL1 (Fig. S3A and B).

The plaque assay provides a comprehensive evaluation of the parasite’s ability to undergo multiple rounds of the lytic cycle, comprised of invasion, replication, and egress events (2, 3). To determine the specific stage of the lytic cycle affected with the loss of TgTKL1 kinase function, we initially performed replication and egress assays. However, we did not see any significant differences in the replication or egress ability of TgTKL1 kinase mutant strain in comparison to wild-type parasites (Fig. S4A and B). Next, we performed host-cell invasion assays using differential staining of extracellular and invaded parasites (red-green invasion assay) (27). The results revealed that TgTKL1-KM strain showed a significant (~65%) decrease in invasion compared to wild-type parasites (Fig. 2C). Importantly, the invasion ability was restored in the TgTKL1-KM-COM strain. These findings suggest that the catalytic activity of TgTKL1 is important for *Toxoplasma* invasion.

In the invasion assay, parasites with defects in the penetration process typically exhibit a decrease in the number of invaded parasites along with a concomitant increase in the number of attached parasites (28). In contrast, specific defects in the host-cell attachment process result in a decrease in the numbers of both attached and invaded parasites. As the TgTKL1-KM parasites did not show an increase in the number of attached parasites, we hypothesized that the loss of kinase function of TgTKL1 specifically impairs host cell attachment.

To determine if TgTKL1 kinase activity is necessary for parasite attachment, we performed attachment assays by treating parasites with mycalolide B, an irreversible inhibitor of actin polymerization (18, 29). *Toxoplasma* tachyzoites treated with this drug are able to secrete micronemal proteins and attach to the host-cell, but cannot penetrate and complete the invasion process, as actin polymerization is critical for parasite motility (30). These assays revealed that, in comparison with the parental strain, TgTKL1-KM parasites showed a significant reduction (~67%) in attachment and that the level of attachment was restored in the TgTKL1-KM-COM strain (Fig. 2D). These results demonstrated that the invasion phenotype observed in TgTKL1-KM parasites is specifically due to impairment of host cell attachment.

### Loss of kinase function of TgTKL1 leads to global changes in *Toxoplasma* gene expression profile

**Fig 3:**
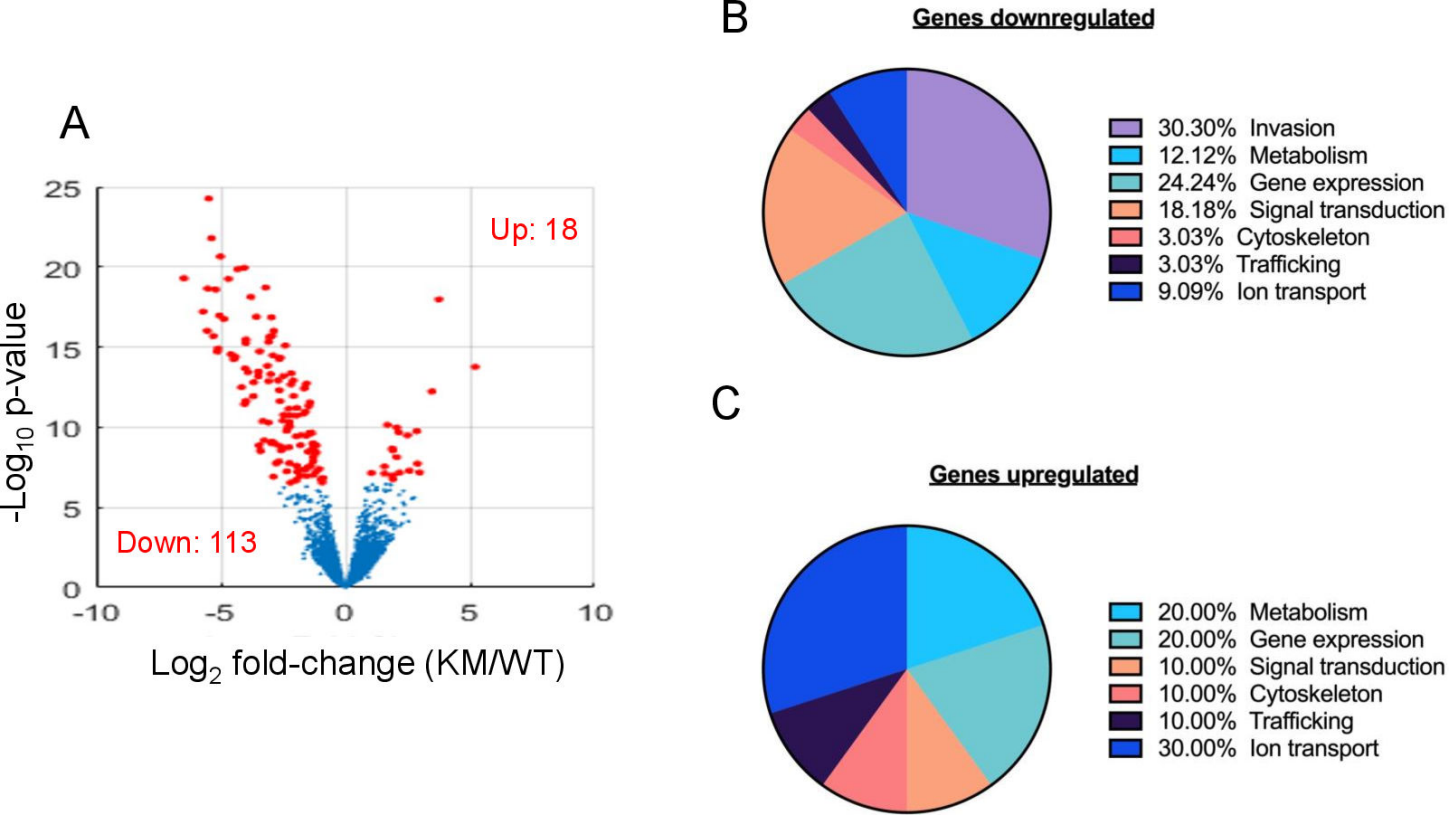
Loss of kinase function of TgTKL1 leads to global changes in the gene expression profile of *Toxoplasma*. (**A**) Volcano plot of statistical significance (−log_10_ *P* value) vs fold change (log_2_), highlighting genes identified as differentially expressed (FDR, ≤0.01) in the TgTKL1-KM strain compared to the parental RHΔKu80 strain. Downregulated genes (*n* = 113) are marked in red on the left, while upregulated genes (*n* = 18) are highlighted in red on the right. (**B and C**) Pie charts showing different categories of genes that are downregulated (**B**) and upregulated (**C**) in TgTKL1-KM strain compared to parental RHΔKu80 strain. Classifications were manually assigned according to known gene functions or putative functions based on conserved domains.

Since TgTKL1 is known to be involved in regulating gene expression in *Toxoplasma*, we wanted to determine if the loss of the kinase activity of TgTKL1 would also lead to alterations in the gene expression profile of the parasite (18). To address this question, we performed RNAseq analysis of wildtype and TgTKL1-KM strains after purification of RNA. The transcriptomic analysis revealed that there were 131 genes (|log2fold change [log2FC] ≥1, false-discovery rate [FDR] ≤1%) that were dysregulated in the kinase mutant compared to wild-type parasites. Among these genes, 113 genes were downregulated, while 18 genes were upregulated in TgTKL1-KM parasites (Fig. 3A; Data Set S1). Subsequently, we manually assigned functional classifications to these genes based on their known or putative functions (Fig. 3B and C). And importantly, a large group of genes that were downregulated are related to parasite invasion (Fig. 3B). These findings, thus, suggest that kinase activity of TgTKL1 is a critical determinant of TgTKL1 function in *Toxoplasma*.

### TgTKL1 catalytic activity is required for efficient microneme protein processing

**Fig 4:**
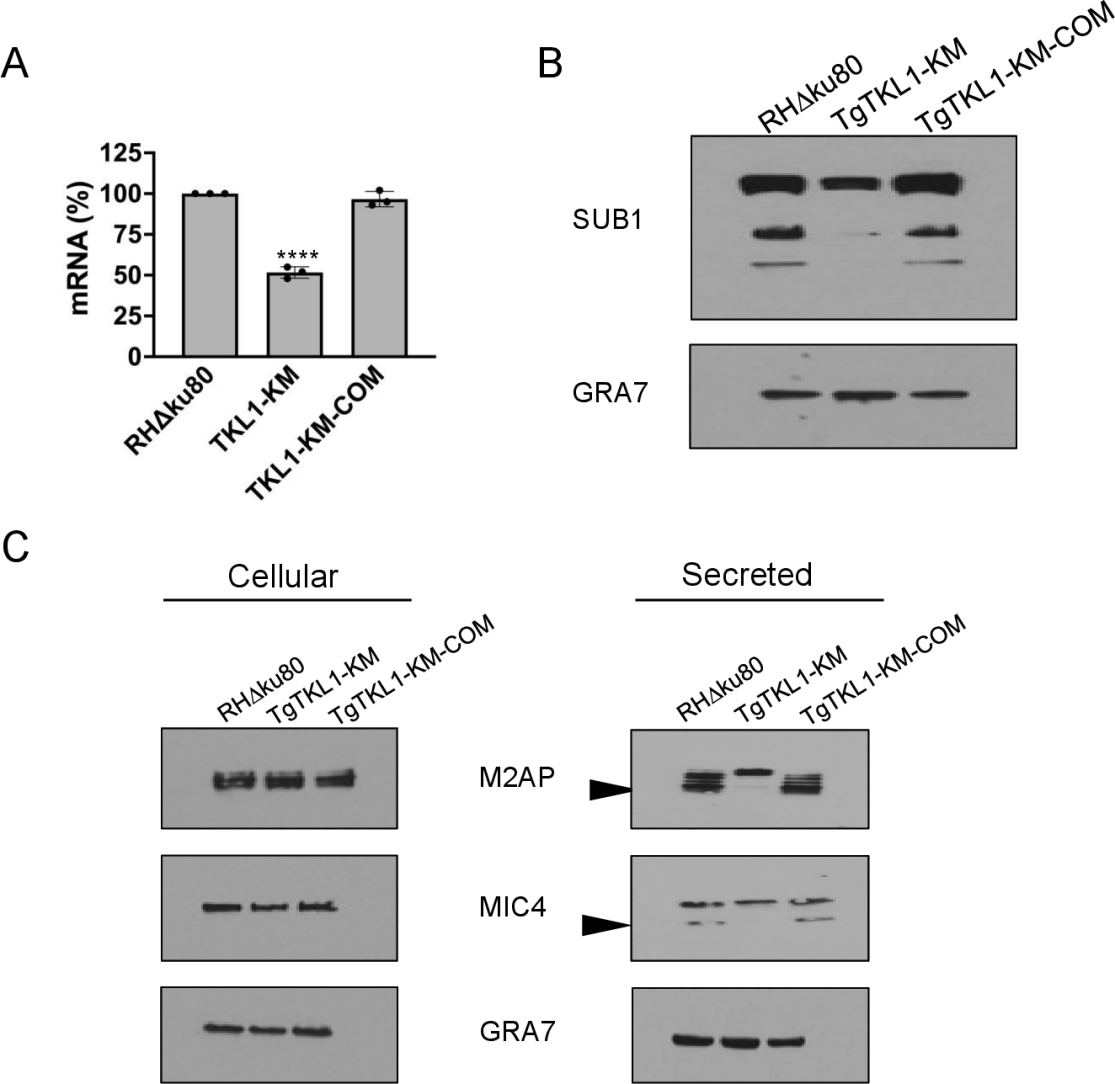
Loss of TgTKL1 activity results in decreased expression of TgSUB1 and defect in microneme processing. (**A**) Quantification of TgSUB1 transcript levels in wild-type (RHΔKu80), kinase mutant (TgTKL1-KM), and complemented (TgTKL1-KM-COM) strains by qRT-PCR. Data represent averages of results from three independent experiments each performed with technical triplicates ± SEM, ****P < 0.0001, one-way ANOVA. (**B**) Representative Western blot image of wild-type (RHΔKu80), kinase mutant (TgTKL1-KM), and complemented (TgTKL1-KM-COM) parasite lysates probed with anti-TgSUB1 antibody. GRA7 is used as the loading control. (**C**) Parasite lysate (cellular fraction) and ESA fractions from wild-type (RHΔKu80), kinase mutant (TgTKL1-KM), and complemented (TgTKL1-KM-COM) strains. ESA fractions were collected from culture supernatants following a 2-min treatment of parasites at 37°C with 1% ethanol to stimulate microneme secretion. GRA7 was used as the loading control for each strain.

Transcriptome analysis revealed downregulation of invasion-related genes in the TgTKL1-kinase mutant in comparison to wild-type parasites. Interestingly, one of these genes is TgSUB1, a protease that has been shown to be essential for the processing of microneme proteins during host-cell invasion (31). Upon secretion of microneme proteins, TgSUB1 traffics to the parasite surface and cleaves secreted micronemal proteins, thereby enhancing their adhesive properties (31). Consequently, any impairment in TgSUB1 expression or secretion will significantly impair host cell attachment of *Toxoplasma*. We first validated the decreased transcript levels of TgSUB1 in the TgTKL1 kinase mutant through qRT-PCR. We observed about 52% decrease in the TgSUB1 mRNA levels in the mutant compared to the parental strain (Fig. 4A). Notably, TgSUB1 transcript levels were restored in the TgTKL1-KM-COM strain, thus suggesting loss of kinase activity TgTKL1 is responsible for this decrease (Fig. 4A). Furthermore, we also assessed TgSUB1 expression at the protein level by immunoblotting. The results showed that there is a substantial reduction (~58%) in TgSUB1 protein levels in TgTKL1-KM compared to the parental strain (Fig. 4B). Importantly, this reduction in the expression of TgSUB1 was restored in the TgTKL1-KM-COM strain (Fig. 4B), suggesting kinase activity of TgTKL1 is required for these gene regulatory events.

Since previous studies have shown that TgSUB1 plays a critical role in *Toxoplasma* invasion (31), we wanted to determine the effect of its decreased expression and secretion in the TgTKL1 kinase mutant. Toward this goal, we initially assessed the expression of micronemal proteins MIC4 and M2AP in the three strains by western blotting. As a control, we also tested expression levels of dense granule protein, GRA7 that is constitutively secreted by the parasite. Interestingly, these assays showed that there is no apparent difference in the expression levels of M2AP and MIC4 among parental, kinase mutant and complemented strains (Fig. 4C). Furthermore, trafficking of these two proteins to the micronemes also appears to be unaffected by the loss of TgTKL1 kinase function (Fig. S5). Subsequently, we induced microneme secretion by treating parasite strains with ethanol, and the secreted fraction was analyzed via western blotting. The findings revealed that the secretion of micronemal proteins MIC4 and M2AP was diminished in TgTKL1-KM parasites compared to wild-type and TgTKL1-KM-COM strains (Fig. 4C; Fig. S6). Additionally, TgTKL1-KM parasites displayed significant defects in the post-exocytosis surface processing of M2AP and MIC4 (Fig. 4C; Fig. S6). These processing events occur after the release of micronemal proteins onto the parasite surface and are essential for host cell attachment (18, 31). Together, these results suggested that the defects in tachyzoite growth observed *in vitro* in TgTKL1-KM parasites are mainly due to the impairment of host cell attachment, resulting from defects in TgSUB1 expression and secretion.

### TgTKL1 kinase activity is essential for parasite virulence in the animal model

**Fig 5:**
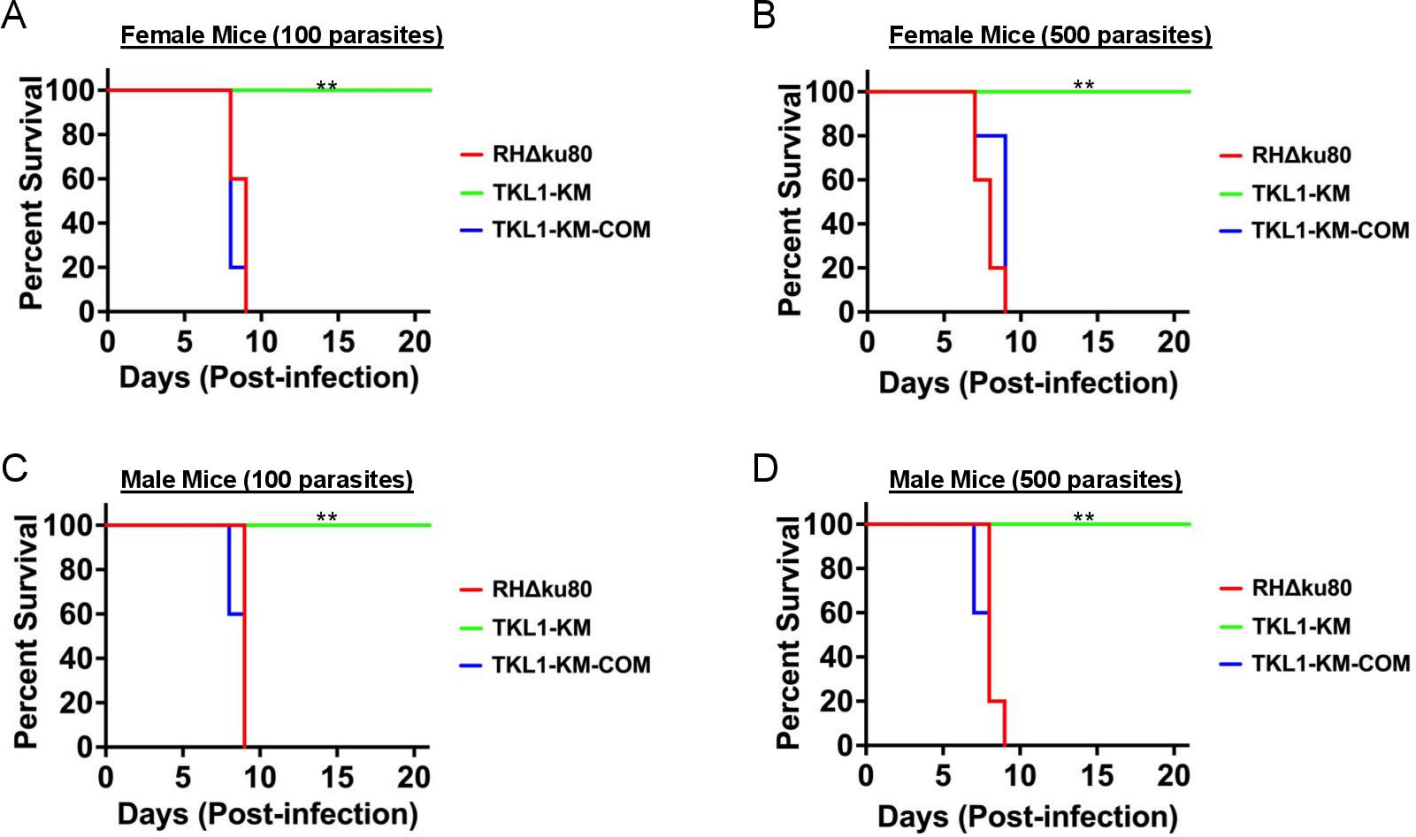
TgTKL1 kinase activity is essential for parasite virulence in the animal model. (**A**) Survival curves for female mice injected with 100 parasites. **P = 0.0015, Log-rank (Mantel-Cox) test (GraphPad Prism). (**B**) Survival curves for female mice injected with 500 parasites. **P = 0.0014, Log-rank (Mantel-Cox) test (GraphPad Prism). (**C**) Survival curves for male mice injected with 500 parasites. **P = 0.0017, Log-rank (Mantel-Cox) test (GraphPad Prism). (**D**) Survival curves for female mice injected with 500 parasites. **P = 0.0016, Log-rank (Mantel-Cox) test (GraphPad Prism). Mice were injected intraperitoneally with 100 or 500 tachyzoites of wild-type (RHΔKu80), kinase mutant (TgTKL1-KM), or complemented (TgTKL1-KM-COM) strains (five male or five female mice for each strain for each dose).

Our studies so far indicated that kinase activity of TgTKL1 is important for *Toxoplasma* invasion. And since previous studies have shown that invasion-related proteins contribute *Toxoplasma* pathogenesis *in vivo* (32) and importantly, that deletion of TgTKL1 results in loss of parasite virulence, we wanted to test the effect of mutation of TgTKL1 kinase domain (18). Toward this goal, we performed survival experiments using mice as an animal model. It is well established that intra-peritoneal inoculation of a single viable *Toxoplasma* tachyzoite leads to the death of the infected mouse within 10 days post-infection (10, 11, 33). Hence, to determine the importance of kinase activity of TgTKL1 in parasite virulence, five male and five female CBA/J mice were infected with either 100 or 500 parasites of wild-type, kinase mutant, or complemented parasites via intraperitoneal injection, and the animals were monitored for survival. Remarkably, all mice infected with either wild-type or complemented parasites died within 10 days of infection (Fig. 5). In contrast, all mice injected with TgTKL1-KM parasites survived. Analysis of serum samples from the survivor mice at 3 weeks post-infection confirmed parasite exposure, with all mice showing seropositivity (Fig. S7). These findings showed that the loss of TgTKL1 kinase activity leads to complete loss of parasite virulence, thus underscoring the significance of the catalytic function of TgTKL1 in *Toxoplasma* virulence*.* Furthermore, since the kinase mutant exhibits loss of virulence in both male and female mice, the results also suggest that contribution of TgTKL1 kinase function to *Toxoplasma* pathogenesis is independent of the sex of the animal.

## DISCUSSION

TgTKL1 is a nuclear kinase in *Toxoplasma* that has been shown to be important for parasite growth *in vitro* and essential for pathogenesis *in vivo* (18). However, the significance of the kinase activity to the overall function of the protein was not known. In this study, we show that TgTKL1 is a true kinase, and its catalytic activity is essential for its function. More specifically, we generated the TgTKL1 kinase mutant through modification of the conserved DFG motif in its kinase domain. This kinase mutant displays defects in parasite growth, host-cell invasion, microneme secretion, and processing seen previously with the TgTKL1 deletion mutant (18). Additionally, the kinase activity of TgTKL1 is also required for its role in regulating gene expression in *Toxoplasma*. More importantly, our work also reveals that the kinase function of this nuclear protein is critical for TgTKL1 role in parasite virulence.

The bioinformatic analysis suggested that TgTKL1 is a true kinase. Through mutation of the conserved DFG motif on the chromosomal copy and functional analysis of the mutant strain, we show that TgTKL1 kinase activity is, indeed, a bona fide kinase. And as TgTKL1 kinase mutant phenocopies TgTKL1 null mutant with respect to parasite growth *in vitro* and virulence *in vivo*, our work demonstrates that the kinase activity of TgTKL1 is critical for its overall function.

It is interesting to note that the TgTKL1 kinase mutant shows changes in gene expression profile similar to the TgTKL1 deletion mutant (18). Of the 131 genes that are dysregulated in the TgTKL1 kinase mutant, a total of 79 genes are also found in the list of genes that are differentially expressed in the TgTKL1 null mutant. Specifically, 69 of 113 genes upregulated and 10 of 18 genes downregulated in the TgTKL1 kinase mutant show similar gene expression pattern in TgTKL1 knockout strain compared to wild-type parasites (18). TgSUB1 is one of the invasion-related genes that are downregulated with the loss of TgTKL1 function, and our findings are in alignment with previous studies that showed that loss of TgSUB1 results in a defect in microneme processing and invasion (31, 34). This also provides a logical explanation for growth defect seen with TgTKL1 kinase mutant as well as the TgTKL1 knockout parasites (18). However, it is quite unlikely that the virulence phenotype exhibited by TgTKL1 kinase mutant is solely due to decreased TgSUB1 expression considering that the TgSUB1 deletion mutant shows some attenuation in virulence but is not avirulent (31). Hence, it is more likely cumulative effect of multiple downregulated genes in the TgTKL1 kinase mutant is contributing to the loss of virulence phenotype.

In addition to the kinase fold TgTKL1 also contains additional domains including EDR1 and RNI domains (Fig. 1B) both of which are predicted to be important for protein-protein interactions. Kinases with EDR1 domain were first described in *Arabidopsis* and are restricted to plants and protozoa (35, 36). Both EDR1 and RNI domains have been predicted to be important for protein-protein interactions (37). However, the contribution and significance of these domains to the overall function of TgTKL1 in *Toxoplasma* still need to be ascertained.

Since TgTKL1 is a nuclear kinase and its kinase activity is important for its function, questions arise with regard to its substrates and signaling pathway mediated by this enzyme. More importantly as TgTKL1 appears to exert its function through regulation of gene expression in *Toxoplasma*, it is feasible that the substrates of TgTKL1 are parasite transcription factors. We anticipate that the precise identification of these substrates will be achieved in the near future using multipronged approaches including proximity-based labeling and quantitative phosphoproteomics that have been successfully employed in *Toxoplasma* (15, 38).

## MATERIALS AND METHODS

### Host cells and parasite cultures

*Toxoplasma gondii* tachyzoites were maintained by serial passage in human foreskin fibroblasts (HFF) within a humidified incubator at 37°C with 5% CO_2_ (19). The standard growth medium comprises Dulbecco’s modified Eagle’s medium (DMEM) supplemented with 10% fetal bovine serum, 2 mM l-glutamine, and 50 µg/mL of penicillin-streptomycin. Parasite propagation, harvest, and purification were performed as previously described (15).

### Expression and purification of recombinant wild-type and mutant TgTKL1 kinase domain

The kinase domain of TgTKL1 was amplified from cDNA of RHΔku80 strain using primers (TKL1.KD.NheI.F and TKL1.KD.NotI.R) and directionally cloned into a bacterial expression vector, pET-28a(+) using restriction enzyme-mediated cloning. The mutation of the DFG motif in the TgTKL1 kinase domain expression construct was carried out using Q5 DNA site-directed mutagenesis kit (New England Biolabs) with primers (DFG.AAA.SDM.F and DFG.AAA.SDM.R). The C-terminal-HIS-tagged wild-type and mutant TgTKL1 expression constructs were then transformed into BL21-Rosetta (DE3) pLysS cells. The induction of recombinant protein was done using IPTG at 37°C. His-tagged recombinant proteins were then purified under native conditions using QIAexpress Ni-NTA fast start kit (Qiagen) according to the manufacturer’s protocol (15).

### Kinase assay

The *in vitro* kinase assay was performed in 96-well plates (100 µL total volume) using ADP-Glo kinase assay kit (Promega) according to the manufacturer’s instructions. Briefly, 2.5 µL of purified recombinant kinase (100 nM) was mixed with the 2.5 µL of 10× kinase buffer, 5 µL of kinase substrate, Myelin basic protein (final concentration 1 mM), 2.5 µL ultra-pure ATP (10 mM stock), and 12.5 µL of water. The reaction was then incubated at 30°C for 30 min. After incubation, 25 µL ADP-Glo reagent (Promega) was added to the reaction mix, and the plate was incubated at room temperature for 40 min. The kinase detection reagent (Promega) 50 µL was then added to the reaction followed by incubation at room temperature. After 60 min incubation, the luminescence was read using plate reader (Synergy H1 from BioTek).

### Immunofluorescence microscopy

Immunofluorescence staining of intracellular parasites was performed according to previously described procedures (19, 39). Primary antibodies included mouse anti-HA antibody (Cell Signaling Technology, Inc.) (6E2, 1:250) and rabbit anti-IMC3 antibody (1:500). The secondary antibodies used included Alexa Fluor 594-conjugated goat anti-rabbit antibody or Alexa Fluor 488-conjugated goat anti-mouse antibody (Molecular Probes) (1:1,000). Slides were viewed using a Zeiss Axio Observer 7 microscope (Carl Zeiss), and digital images were captured with an Axiocam 506 mono charge-coupled-device camera using Axiovision software (19).

### Generation of TgTKL1 kinase mutant and complemented strains

To generate the TgTKL1-kinase mutant strain, a single-guide RNA (gRNA) sequence targeting the DFG motif of the TgTKL1 kinase domain was introduced in the CRISPR-Cas9 plasmid (40) using Q5 DNA site-directed mutagenesis (New England Biolabs). The reaction was performed according to the manufacturer’s protocol, and the correct modification was verified by Sanger sequencing. The CRISPR-Cas9-sgRNA plasmid, along with the repair template (made of DNA oligos) containing codons for AAA motif in place of DFG with 40 bp flanking regions, was subsequently electroporated into RHΔKu80 strain using Nucleofector (25). The transfected parasites were then added onto HFF monolayer until the lysis of the monolayer. The freshly egressed parasites were then harvested, purified, and cloned by limiting dilution. The mutation of the DFG motif in the TgTKL1 gene of the clones was verified by the purification of genomic DNA, PCR, and sequencing.

The complementation of TgTKL1-kinase mutant clone was performed as described previously (18). Briefly, cosmid clone PSBLW20 containing TgTKL1 gene was transfected into the TgTKL1 kinase mutant strain followed by culturing in the presence of phleomycin (41). The drug-resistant parasites were then cloned by limiting dilution and verified by sequencing.

TgTKL1 was endogenously tagged at the C terminus using the methods described in the previous study (18). Briefly, genomic DNA was purified from RHΔKu80 parasites and used as a template to amplify fragments upstream of the stop codon of TgTKL1. The PCR product was then cloned into the PacI site of plasmid pLIC.HA3.DHFR using an In-Fusion HD cloning kit (Clonetech). The resulting plasmid was linearized and electroporated into RHΔKu80 or TgTKL1-KM or TgTKL1 knockout parasites. The transfected parasites were cultured in the presence of pyrimethamine (1 µM) to select for drug-resistant parasites, and single clones were then isolated by limiting dilution.

### Plaque assays

Intracellular parasites were harvested, syringe filtered, and subsequently added onto a confluent monolayer of HFF cells in a 12-well plate (500 or 1,000 tachyzoites per well). The plates were then incubated at 37°C undisturbed for 6 days. The plates were then washed with PBS, fixed with methanol, stained with 2% crystal violet, and imaged using Molecular Imager Gel Doc XR system (Bio-Rad) with Image Lab software. The plaques were then outlined manually, and the size was determined using ImageJ. A total of three independent experiments were performed to determine the mean plaque sizes.

### Invasion assays

Purified tachyzoites were inoculated onto HFFs plated on 12 mm coverslips in 24-well plates (5 × 10^6^ parasites/well) and incubated at 37°C for 30 min. The plates were then washed with PBS to remove most of the non-invaded parasites. Subsequently, the coverslips were fixed, blocked, and stained with rat anti-SAG1 antibody (1:500) for 1 h. After incubation, the slides were washed, permeabilized with 0.01% Triton X-100, and stained with rabbit anti-M2AP antibody (1:500). The slides were further washed and stained with secondary antibodies, namely, Alexa Fluor 594-conjugated goat anti-rat antibody (Molecular Probes) and Alexa Fluor 488-conjugated goat anti-rabbit antibody (Molecular Probes) (1:1,000). After 1 h, the slides were washed and mounted with Vectashield (with DAPI [4′,6-diamidino-2-phenylindole]). Parasites displaying both red and green fluorescence were identified as extracellular (attached), while those exhibiting only green fluorescence were categorized as intracellular (invaded) (42). Fifteen fields were randomly selected per coverslip, and images were captured using a 63× objective, and the total numbers of intracellular parasites and host-cell nuclei were counted.

### Attachment assays

Purified tachyzoites were treated with 3 µM mycalolide B (Enzo Life Sciences) for 10 min, followed by three washes with DMEM–10% fetal bovine serum (FBS). The parasites were then added onto HFF monolayer in an eight-well chamber slide (2 × 10^6^ parasites per well) and incubated at 37°C for 30 min. Slides were then washed, fixed, blocked, and stained with mouse anti-SAG1 antibody. Following another round of washing, slides were stained with a secondary antibody, Alexa Fluor 594-conjugated goat anti-mouse antibody (Molecular Probes). After an additional hour of incubation, the slides were washed and mounted using Vectashield (with DAPI). Images of 15 random fields of view within each well were then performed using Zeiss wide-field fluorescent microscope. The number of attached parasites and host cell nuclei were then enumerated, and results from three independent experiments were included in the statistical analysis (18, 39).

### Microneme secretion assays

Ethanol-induced microneme secretion was performed according to previously described protocols (32). Briefly, freshly egressed parasites were harvested, filtered, and resuspended in 200 µL (5 × 10^7^ parasites) of DMEM supplemented with 20 mM HEPES. A 100 µL volume (2.5 × 10^7^ parasites) of parasite suspension was then added to 100 µL of preheated (37°C) DMEM containing 2% ethanol in Eppendorf tubes, and excreted/secreted antigen (ESA) induction was carried out for 2 min at 37°C. The tubes were then immediately placed on ice for 2 min, followed by centrifugation at 1,000 × *g* for 10 min at 4°C. A total of 140 µL of the supernatant was collected and subjected to a second centrifugation, after which 100 µL was removed and mixed with 5× SDS-PAGE sample buffer. The ESA fraction samples were then heated at 95°C for 5 min, cooled on ice for 2 min, and resolved on a 4%–20% gradient gel (Bio-Rad, Hercules, CA). Subsequently, proteins were transferred from the gel to a nitrocellulose membrane using a wet-transfer apparatus (Bio-Rad, Hercules, CA) at 100 V for 2 h. After blocking with 5% (wt/vol) skim milk powder in Tris-buffered saline (TBS), membranes were probed with rabbit anti-MIC4 (1:5,000), rabbit anti-M2AP (1:2,000), and mouse anti-GRA7 (1:5,000) for 1 h. After washing, membranes were incubated with either horseradish peroxidase (HRP)-conjugated goat anti-rabbit IgG (Sigma) or HRP-conjugated goat anti-mouse IgG (Sigma). Following additional washing steps, membranes were treated with SuperSignal West Femto chemiluminescent substrate (Pierce Chemical) and imaged using Flourchem Protein Simple.

### Replication assay

Toxoplasma replication assay was performed according to previously described procedures (19). Briefly, to assess the parasite doubling time, freshly egressed parasites were inoculated into confluent HFF monolayers in 12-well plates and allowed to invade for 2 h. The monolayers were then washed three times with medium to remove uninvaded parasites and incubated at 37°C. At 30 h post-infection, the cells were fixed with methanol and stained using Diff-Quik (Dade-Behring) according to the manufacturer’s instructions. For each treatment, at least 100 vacuoles from three biological replicates were assessed for the number of parasites per vacuole.

### Ionophore-induced egress assay

The efficiency of egress after calcium ionophore treatment was determined using established protocols (43). Briefly, freshly harvested parasites were added to a 24-well plate containing confluent HFFs at a multiplicity of infection (MOI) of 1 and were incubated at 37°C for 30 h. To induce egress, intracellular parasites were washed with warm PBS, incubated at 37°C for 2 min in Hanks’ balanced salt solution (HBSS) containing 1 µM calcium ionophore A23187, and fixed with 100% methanol. To visualize intact and lysed vacuoles, the cultures were stained using Diff-Quik (Dade-Behring) according to the manufacturer’s instructions. Percent egress was determined by dividing the number of lysed vacuoles by the total number of vacuoles for a sample.

### RNA sequencing and differential gene expression analysis

RNA sequencing was performed according to previously published protocols with some modifications (18, 19). Intracellular parasites, 24 h post-infection were harvested by scraping monolayer in PBS, passed through 21-, 23-, and 25-gauge syringe needles. The parasites were then filtered and pelleted at 1,000 *g* for 10 min. Total RNA from the pelleted parasites was isolated using the RNeasy kit (Qiagen). The quality of total RNA samples was verified by using a BioAnalyzer (Agilent), followed by digestion with DNase I (NEB). rRNA was removed using the Ribo-Zero rRNA removal kit (human/mouse/rat; Illumina). Sequencing libraries were then generated using the TruSeq RNA sample prep kit (v2; Illumina) according to the manufacturer’s protocol. Libraries were amplified using the TruSeq cluster kit (v3; Illumina) and subjected to 50 bp single-end sequencing with the Illumina HiSeq 2000 system. Sequencing reads were aligned to the *Toxoplasma* GT1 reference genome (www.toxodb.org) using the STAR software package (v.2.7.1a, with default settings). Filtered and normalized gene expression levels were calculated from the aligned reads using HTSeq v.0.13.5. Differentially expressed genes were identified by linear modeling and Bayesian statistics using the limma package for R v.3.49.1. Quantitative RT-PCR was performed using Fast SYBR green master mix (Thermo, Fisher), and data were analyzed as described in previously and normalized to alpha-tubulin levels (29). The primers used for qRT-PCR analysis are listed in Table S1. Both RNA sequencing and qRT-PCR analysis were performed on RNA samples obtained from three independent experiments.

### *In vivo* virulence assays

All laboratory animal work in this study was carried out in accordance with policies and guidelines specified by the Office of Laboratory Animal Welfare, the US Department of Agriculture, and the American Association for Accreditation of Laboratory Animal Care (AAALAC). The University of Michigan Committee on the Use and Care of Animals (IACUC) approved the animal protocol used for this study (Animal Welfare Assurance A3114-01, protocol no. PRO00008638). Six-week-old female or male CBA/J mice were intraperitoneally injected with TgTKL1-WT, TgTKL1-KM, or TgTKL1-KM-CO parasites. To verify the viability of the injected parasites, an equivalent number of parasites from the same preparation used for mouse injections were inoculated onto an HFF monolayer for plaque assays immediately post-injection. Seroconversion of all surviving mice was verified through western blot analysis using serum collected 3 weeks post-infection and RHΔKu80 parasite lysate (18, 19).

### Dot-blot assay

Parasite (RHΔku80) lysate was heated at 100°C for 5 min in SDS–PAGE sample buffer with 2% 2-mercaptoethanol and blotted onto nitrocellulose membrane. After blocking with 10% (wt/vol) skim milk powder in PBS, membranes were treated with sera collected from survivor mice for 1 h. Membranes were then washed and incubated with anti-mouse secondary antibody conjugated to horseradish peroxidase. After washing, membranes were treated with SuperSignal West Femto chemiluminescent substrate (Pierce Chemical) and imaged using Flourchem Protein Simple.

## SUPPLEMENTAL MATERIAL

Data Set S1 msphere.00779-24-s0001.xlsx List of genes dysregulated in TgTKL1 kinase mutant strain.

Fig. S1 msphere.00779-24-s0002.tiff Sequence alignment, predicted structure, and SDS-PAGE gels of TgTKL1 kinase domain.

Fig. S2 msphere.00779-24-s0003.tiff Immunofluorescence analysis of TgTKL1-KO strains transformed with construct used for complementing TgTKL1 kinase mutant parasites.

Fig. S3 msphere.00779-24-s0004.tiff TgTKL1 kinase mutant shows defect in *in vitro* propagation similar to TgTKL1 null mutant.

Fig. S4 msphere.00779-24-s0005.tiff Loss of TgTKL1 kinase function does not affect *Toxoplasma* replication or egress.

Fig. S5 msphere.00779-24-s0006.tiff Microneme trafficking is not altered in TgTKL1-KM parasites.

Fig. S6 msphere.00779-24-s0007.tiff TgTKL1 kinase function is important for microneme secretion and processing.

Fig. S7 msphere.00779-24-s0008.tiff Dot blot assays confirming seropositivity of mice that survived TgTKL1-KM infection.

Primer List msphere.00779-24-s0009.pdf List of primers used in this study.

Supplemental Legends msphere.00779-24-s0010.docx Legends for supplemental material.

ASM does not own the copyrights to Supplemental Material that may be linked to, or accessed through, an article. The authors have granted ASM a non-exclusive, world-wide license to publish the Supplemental Material files. Please contact the corresponding author directly for reuse.
