# Supplementary figures and images for: Kinase function of TgTKL1 is essential for its role in Toxoplasma propagation and pathogenesis
Source: mSphere. 2024 Oct 30;9(11):e00779-24. doi: 10.1128/msphere.00779-24 (PMC11580469; doi:10.1128/msphere.00779-24)

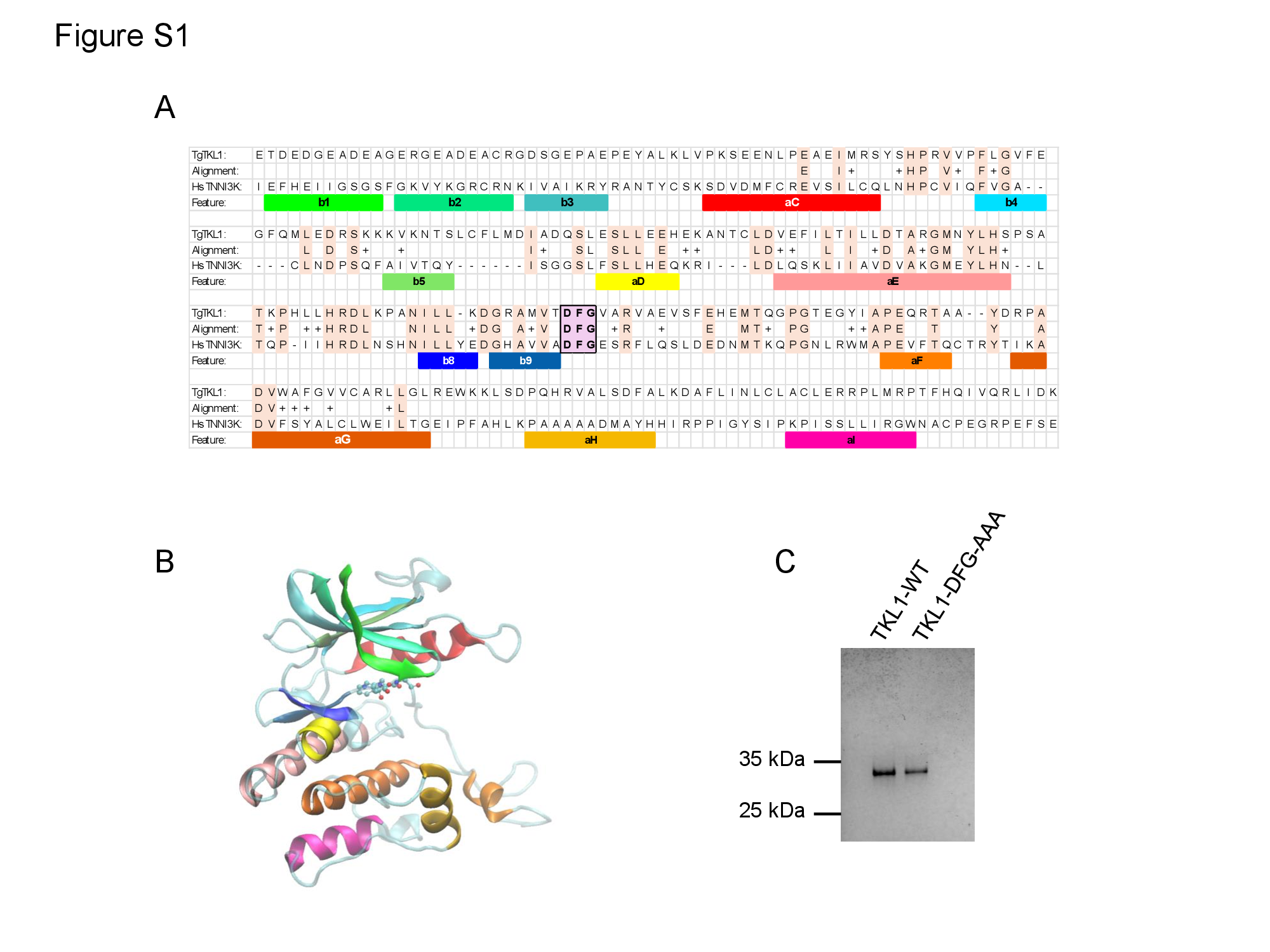

Supplement: Fig. S1 — Sequence alignment, predicted structure, and SDS-PAGE gels of TgTKL1 kinase domain. [file msphere.00779-24-s0002.tiff]

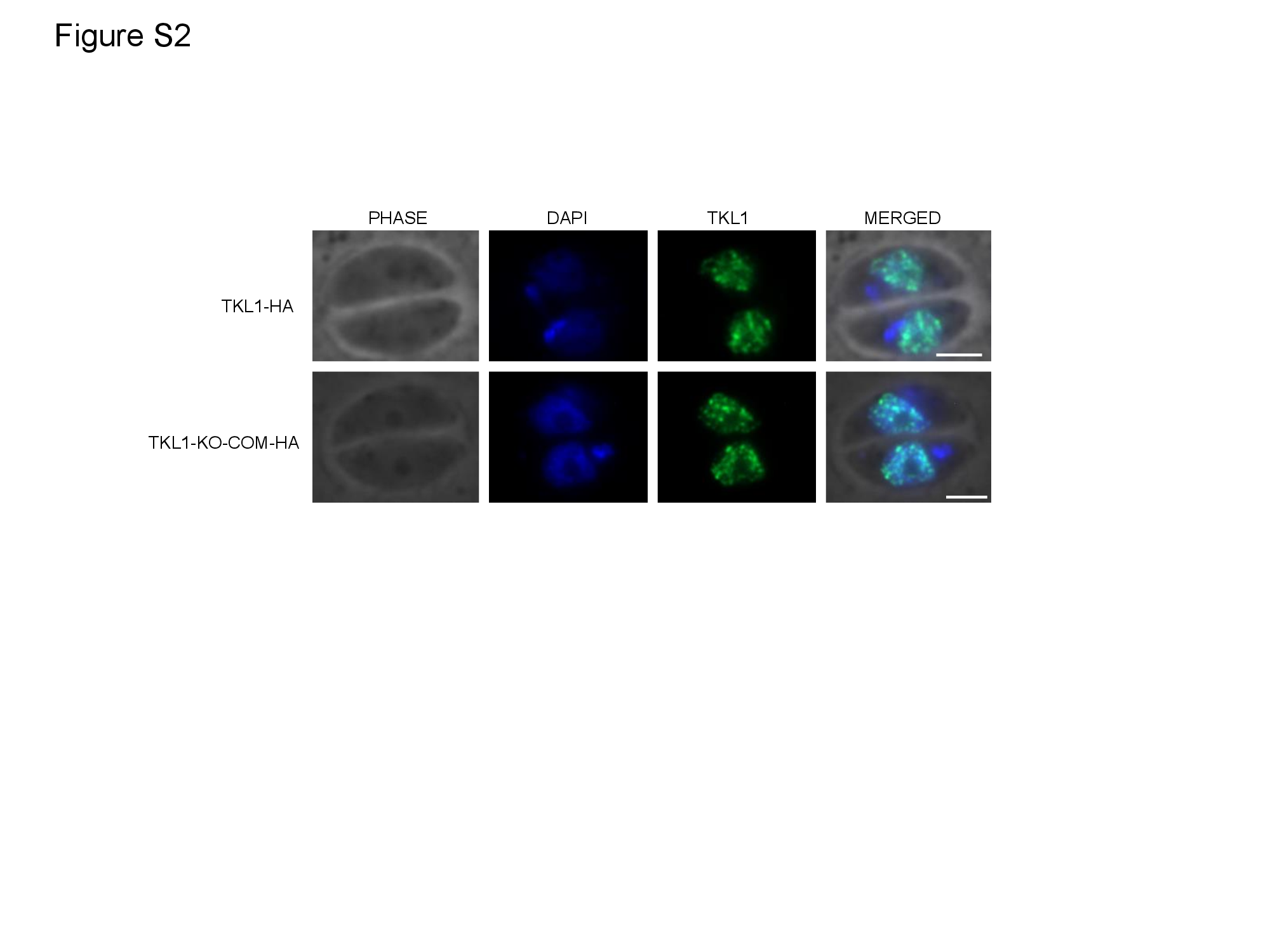

Supplement: Fig. S2 — Immunofluorescence analysis of TgTKL1-KO strains transformed with construct used for complementing TgTKL1 kinase mutant parasites. [file msphere.00779-24-s0003.tiff]

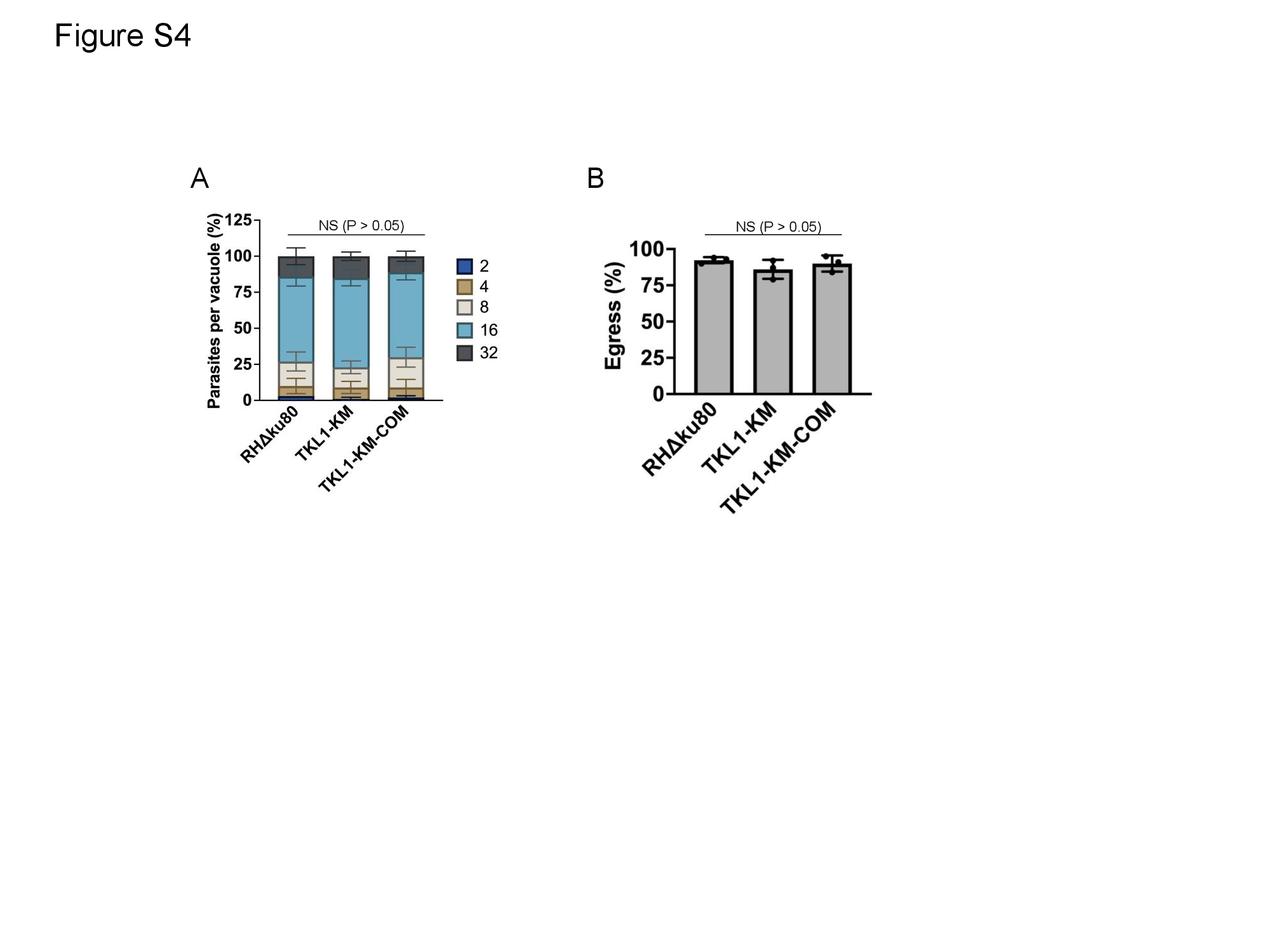

Supplement: Fig. S4 — Loss of TgTKL1 kinase function does not affect Toxoplasma replication or egress. [file msphere.00779-24-s0005.tiff]

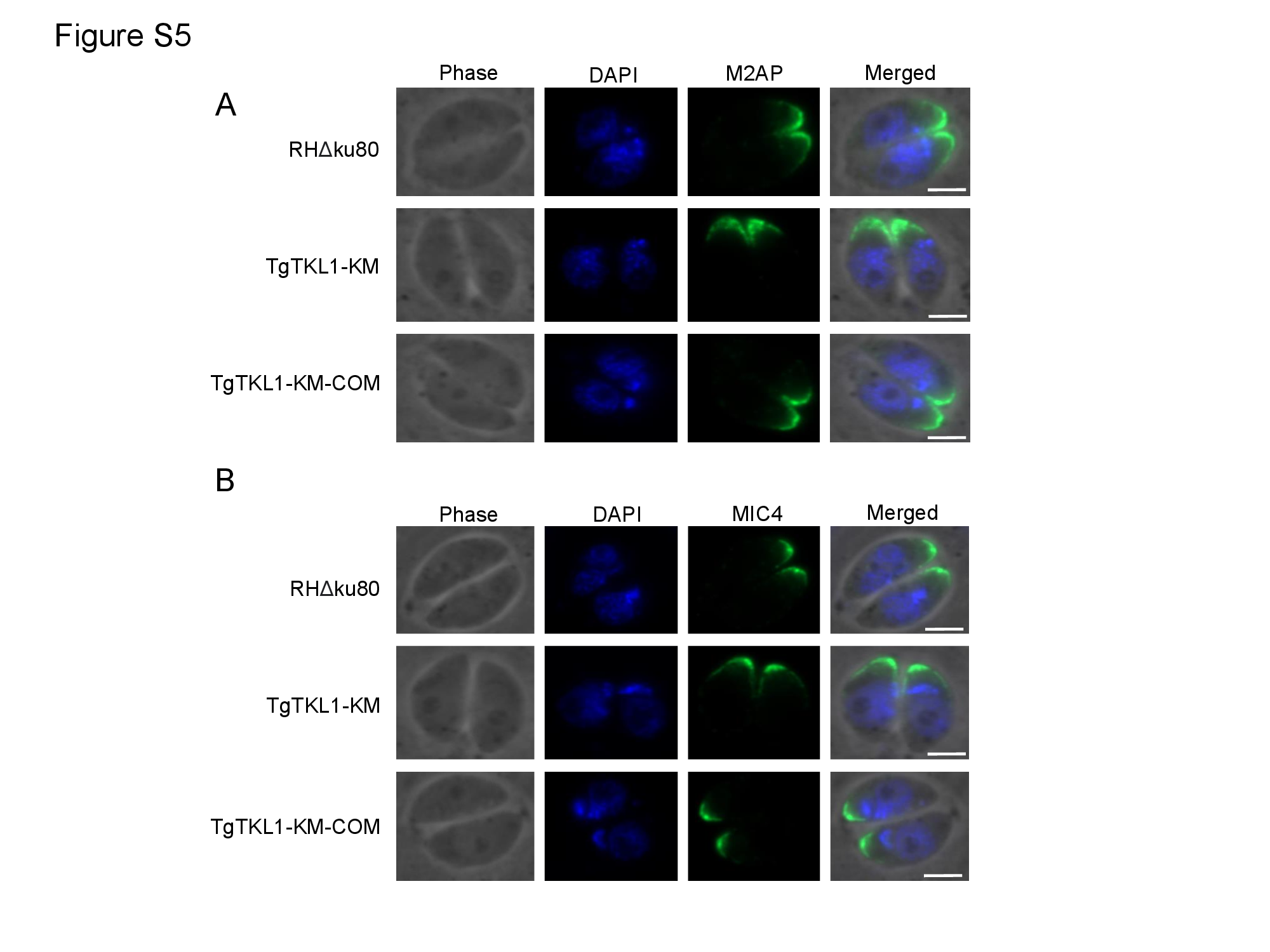

Supplement: Fig. S5 — Microneme trafficking is not altered in TgTKL1-KM parasites. [file msphere.00779-24-s0006.tiff]

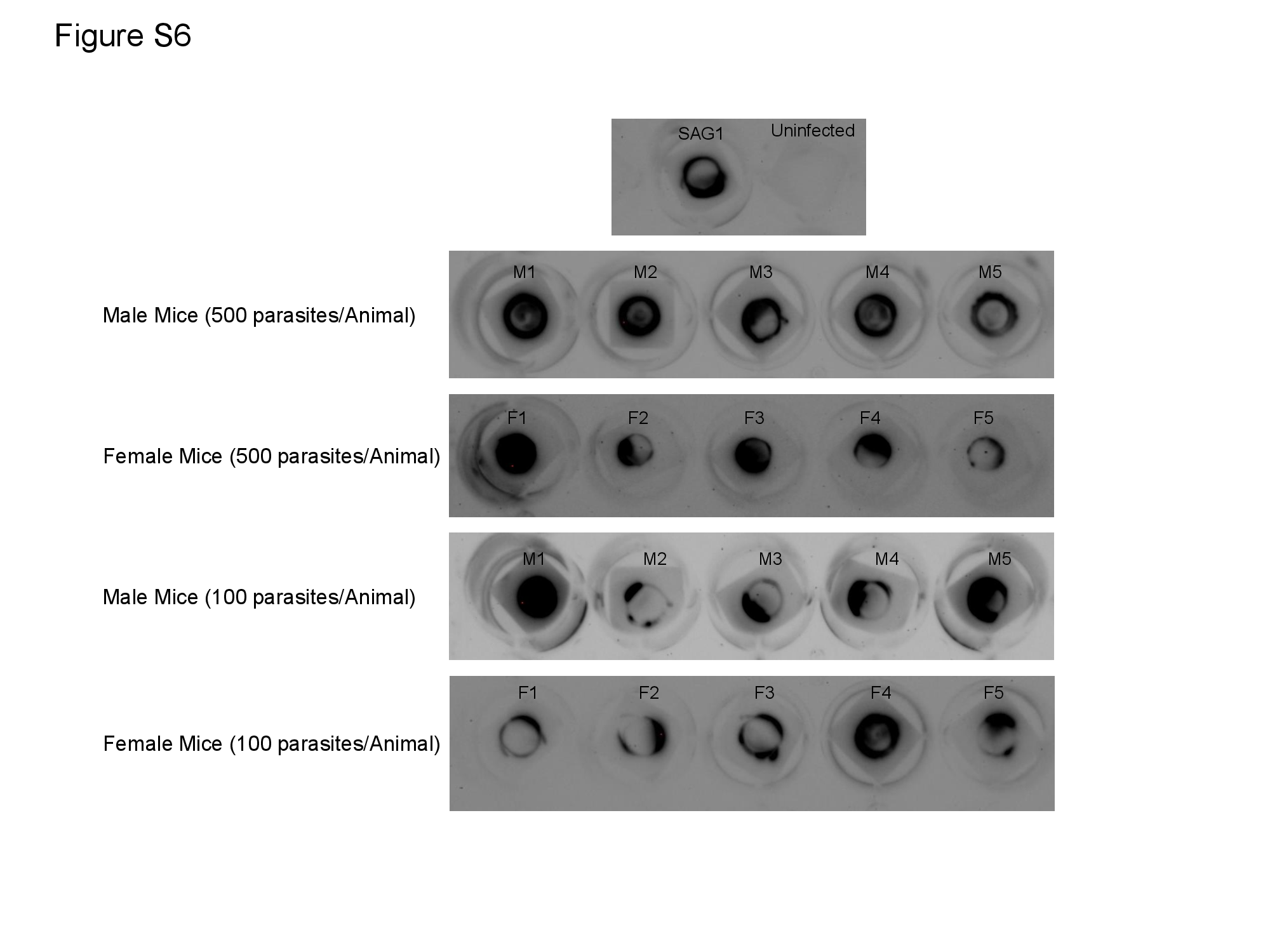

Supplement: Fig. S7 — Dot blot assays confirming seropositivity of mice that survived TgTKL1-KM infection. [file msphere.00779-24-s0008.tiff]
