# Supplementary material for: Kinase function of TgTKL1 is essential for its role in Toxoplasma propagation and pathogenesis
Source: mSphere. 2024 Oct 30;9(11):e00779-24. doi: 10.1128/msphere.00779-24 (PMC11580469; doi:10.1128/msphere.00779-24)
Supplement: Primer List — List of primers used in this study. [file msphere.00779-24-s0009.pdf]

Table S1. List of primers used in this study.

S. No	Name	Sequence (5' to 3')
1	TKL1.KD.SG3.F	gacttcggtgGTTTTAGAGCTAGAAATAGC
2	TKL1.KD.SG3.R	cgtcaccatcAACTTGACATCCCCATTTAC
3	TKL1.KD.RT.F	gttttcttttgcaagtggacatttttgttttcagGATGGAAGAGCGATGGTGACGGCAGCGGCAGTGGCGCGCGTCGCGGAAGTTTCGTTCGAACATGAGATGACGCAGG
4	TKL1.KD.RT.R	CCTGCGTCATCTCATGTTCGAACGAAACTTCCGCGACGCGCGCCACTGCCGCTGCCGTCACCATCGCTCTTCCATCctgaaaacaaaaatgtccacttgcaaaagaaaac
9	DFG.AAA.SDM.F	ccgccGTGGCGCGCGTCGCG
10	DFG.AAA.SDM.R	cggcCGTCACCATCGCTCTTCCATCCTTG
11	TKL1.KD.NheI.F	actgGCTAGCGTGTCTGAGGAAGAAGAGCGA
12	TKL1.KD.NotI.R	actgGCGGCCGCGTTCTCTGCGTGGAGAAGCGG
13	TUBA1.F	GCATGATCAGCAACAGCACT
14	TUBA1.R	GAGAGCAGCCAAATCCTCAC
15	SUB1.F	TATGCCGATGACGACATCCG
16	SUB1.R	ATCCACTAAAACCACGCCAC
