# Supplementary material for: Kinase function of TgTKL1 is essential for its role in Toxoplasma propagation and pathogenesis
Source: mSphere. 2024 Oct 30;9(11):e00779-24. doi: 10.1128/msphere.00779-24 (PMC11580469; doi:10.1128/msphere.00779-24)
Supplement: Supplemental Legends — Legends for supplemental material. [file msphere.00779-24-s0010.docx]

**Legends for supplementary material:**

**Figure S1.** (A) The amino acid sequence of the TgTKL1 kinase domain (residues 2620 to 2885) was aligned with the kinase domain of *Homo sapiens* Troponin I-Interacting Kinase (TNNI3K). Identical residues are highlighted in tan. The sign, “+” indicates alignment of residues with similar chemical properties. The sequences that correspond to key structural features are shown as colored bars: anti-parallel beta sheets (b1-b5, b8, b9) and alpha helices (aC-aI). (B) Predicted structure of TgTKL1 kinase domain showing N and C-lobes and highlighted location of the activation loop motif, DFG (shown with atom level representation). Each beta sheet and alpha helix is represented with a different color. Loops and turns are shown in light blue. The structure of TgTKL1 was built using Modeller, with 4YFF (structure of the protein kinase domain of Troponin I-Interacting Kinase) as the template. (C) Purified recombinant wildtype and mutant version of TgTKL1 kinase domain. The samples were run on SDS-PAGE gel, stained with 0.1% Coomassie blue R250 (Sigma Aldrich) and imaged with FluorChem E system (ProteinSimple).

**Figure S2**. Immunofluorescence analysis of TgTKL1-KO strains transformed with construct used for complementing TgTKL1 kinase mutant parasites. The top panel shows TgTKL1-HA strain, and the IFA was performed using anti-HA antibody. DAPI is used as a marker for the nucleus . Scale bar, 2 μm.

**Figure S3**. TgTKL1 kinase mutant shows defect in *in vitro* propagation similar to TgTKL1 null mutant*.* (A) Plaque assay examining the growth of wild-type (RHΔKu80), kinase mutant (TgTKL1-KM), null mutant (TgTKL1-KO) and TgTKL1 kinase mutant complemented (TgTKL1-KM-COM) strains in HFF cells. Plaques are visible as clear zones on the background of a crystal violet stained HFF monolayer. (B) Quantification of plaque area sizes of wild-type, TgTKL1-KM, TgTKL1-KO and TgTKL1-KM-COM strains. n=3, data represent mean ± standard deviation. *P < 0.001, ns = not significant, One-way ANOVA followed by students’ *t-*test (GraphPad Prism).

**Figure S4.** Loss of TgTKL1 kinase function does not affect *Toxoplasma* replication or egress. The wildtype (RHΔKu80), kinase mutant (TgTKL1-KM) and complemented (TgTKL1-KM-COM) parasites were subjected to doubling (A) and ionophore induced egress (B) assays. Data are compiled from three independent experiments and error bars represent standard deviations. NS = Not significant, One-way ANOVA using GraphPad Prism.

**Figure S5**. Microneme trafficking is not altered in TgTKL1-KM parasites. IFA of M2AP (A), MIC4 (B) in wildtype, TgTKL1-KO, and TgTKL1-KM-COM parasites was performed. Both proteins properly localized to the apical region of the parasite, suggesting that intracellular trafficking is not TgTKL1 dependent. Scale bar, 2 μm.

**Figure S6.** TgTKL1 kinase function is important for microneme secretion and processing. (A) Quantification of relative total amounts of micronemal proteins (M2AP and MIC4) secreted in wildtype (RHΔku80), kinase mutant (TgTKL1-KM), and complemented (TgTKL1-KM-COM) strains following treatment with ethanol. The constitutively secreted dense granule GRA7 protein was used as a control. The ratio of total signals from all the bands of each microneme protein to the GRA7 signal was determined for each of the strains, and the ratio obtained with the RHΔku80 clone was set at 100%. The quantification of signal was performed using a FluorChem E system (ProteinSimple). Data were compiled from results from three independent experiments, and error bars represent standard deviation, P < 0.05, One-way ANOVA using GraphPad Prism. (B) Quantification of relative levels of microneme processing of M2AP and MIC4 in ethanol-induced wildtype (RHΔku80), kinase mutant (TgTKL1-KM), and complemented (TgTKL1-KM-COM) strains. The constitutively secreted GRA7 was used as a control. The ratio of the processed band signal of each of the microneme proteins to the GRA7 signal was determined, and the ratio obtained for RHΔku80 was set at 100%. The quantification of signal was performed using a FluorChem E system (ProteinSimple). Data were compiled from results from three independent experiments, and error bars represent SD. * P < 0.05, One-way ANOVA using GraphPad Prism.

**Figure S7.** Dot blot assays confirming seropositivity of mice that survived TgTKL1-KM infection. The top panel shows the positive (SAG1 antibody) and negative controls (serum from uninfected mouse). The bottom four panels show parasite lysates probed with sera from survivor mice. The assays were performed in 24-well plates as described in materials and methods and imaged using Flourchem Protein Simple.

**SUPPLEMENTAL DATA LEGENDS**

**1. Supplemental table S1.** List of primers used in this study.

**2. Supplemental data set S1.** List of genes dysregulated in TgTKL1 kinase mutant strain.
